# Supplementary material for: Hypoxic Human Microglia Promote Angiogenesis Through Extracellular Vesicle Release
Source: Int J Mol Sci. 2024 Nov 21;25(23):12508. doi: 10.3390/ijms252312508 (PMC11641038; doi:10.3390/ijms252312508)
Supplement: Supplementary file 1 [file ijms-25-12508-s001.zip › ijms-3334242-supplementary.pdf]

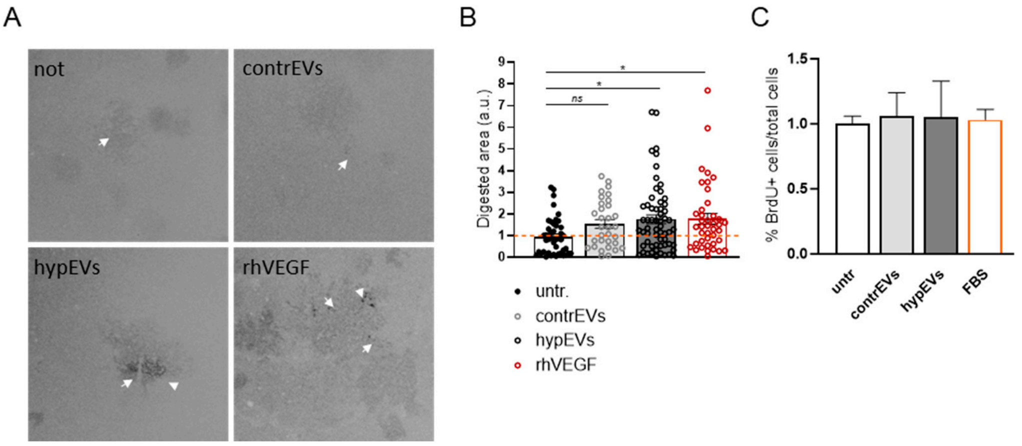

Supplementary Figure S1. Effects of microglial EVs on endothelial matrix digestion and proliferation. A. Representative images of the FITC-gelatin degradation assay. HBEC-5i cells were seeded onto FITC-gelatin coated coverslips, and treated with EVs, or with rhVEGF for 24 h. B. Quantification of the black (digested) area (indicated by the white arrows), was performed with ImageJ. C. Results of the *in vitro* proliferation assay performed with HBEC-5i cells, incubated or not with EVs, or cultured in the presence of 10% FBS, for 6 h. Histograms represent the relative percentage of BrdU$^+$ cells over the total number of cells, compared to the unstimulated control. Bars represent mean ± SEM. N=3. Kruskal-Wallis test was applied. *p<0.05.

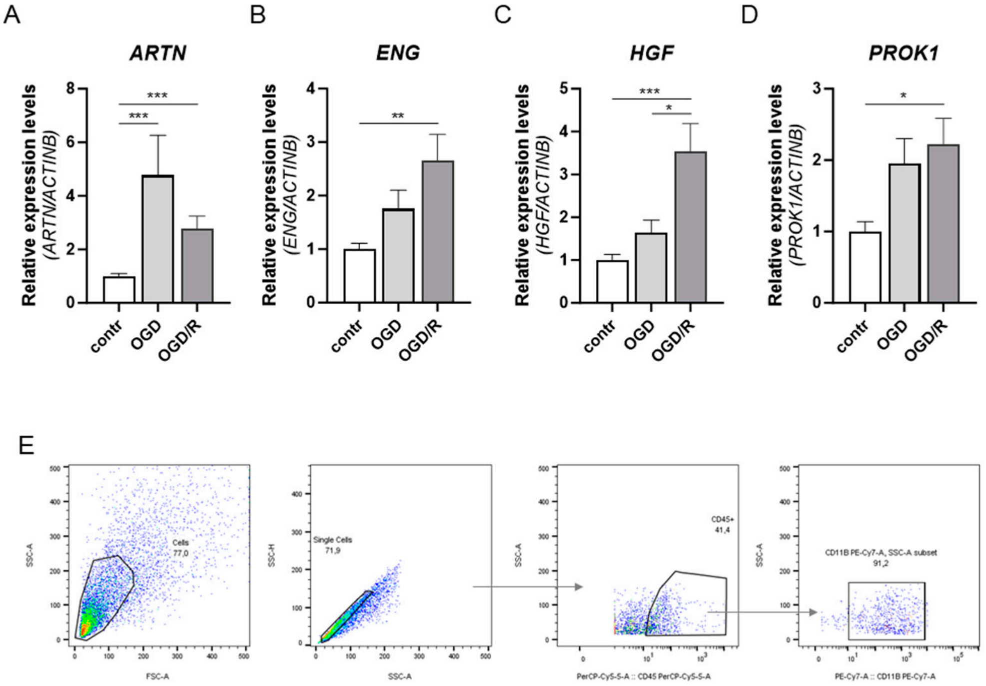

Supplementary Figure S2. qRT-PCR analysis of pro-angiogenic gene expression in HMC-3 cells and microglia isolation. A-D. Gene expression of the identified pro-angiogenic markers in HMC-3 cells exposed to OGD/R, evaluated by qRT-PCR. Bars represent mean ± SEM and normalised to the housekeeping genes *ACTB*. N=3. Kruskal-Wallis test was applied *p<0.05, **p<0.01,***p<0.001. E. Representative panels relative to the flow cytometric analysis of primary microglial cells isolated from healthy adult mice. The panels and arrows represent the gating strategy applied to the CD11b$^+$ cell fraction obtained from the isolation procedure, validating the purity of CD45$^+$CD11b$^+$.

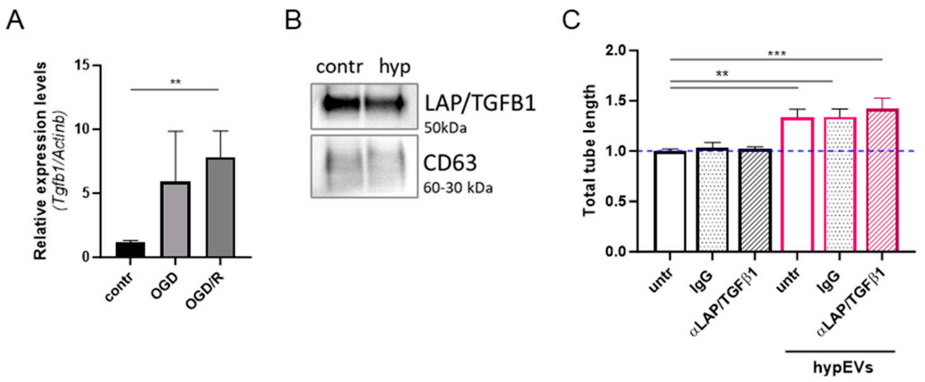

Supplementary Figure S3. Assessing the involvement of the TGF-β1 pathway in the hypEVs pro-angiogenic mechanism. A. Expression levels of *Tgfb1* in primary microglia exposed to the OGD/R protocol, as quantified by qRT-PCR. Expression levels are normalised to the housekeeping gene *Actb.* Bars represent mean ± SEM. N=3. Kruskal-Wallis test was applied $^{**}p<0.01$. B,C. Western blot of EV lysates, showing the expression of LAP/TGF-β1 in purified EV samples. D. Quantification of the total tube length for the tube formation assay performed on HBEC-5i. Cells were treated with hypEVs pre-incubated or not with anti-LAP/TGF-β1 neutralising antibody, or with an isotype control. Tubes were imaged after 6h and the analysis was performed using the Angiogenesis Analyzer tool for ImageJ. N=3. Kruskal-Wallis test was applied. $^{**}p<0.01$, $^{***}p<0.001$.
